# Quantitative Assessment of the Influence of TP63 Gene Polymorphisms and Lung Cancer Risk: Evidence Based on 93,751 Subjects

**DOI:** 10.1371/journal.pone.0087004

**Published:** 2014-01-23

**Authors:** Liang Zhang, Xiao-Feng Wang, Yu-Shui Ma, Qing Xia, Feng Zhang, Da Fu, Yi-Chao Wang

**Affiliations:** 1 Department of Orthopaedics, Zhongshan Hospital, Fudan University, Shanghai, People’s Republic of China; 2 Department of Nuclear Medicine, Shanghai 10th People’s Hospital, School of Medicine, Tongji University, Shanghai, People’s Republic of China; 3 Institute of Health Sciences, Shanghai Institutes for Biological Sciences, Chinese Academy of Sciences/Shanghai Jiao Tong University School of Medicine, Shanghai, People’s Republic of China; University General Hospital of Heraklion and Laboratory of Tumor Cell Biology, School of Medicine, University of Crete, Greece

## Abstract

**Background:**

Several genome-wide association studies on lung cancer (LC) have reported similar findings of a new susceptibility locus, 3q28. After that, a number of studies reported that the rs10937405, and rs4488809 polymorphism in chromosome 3q28 has been implicated in LC risk. However, the studies have yielded contradictory results.

**Methods:**

PubMed, ISI web of science, EMBASE and the Chinese National Knowledge Infrastructure databases were systematically searched to identify relevant studies. Data were abstracted independently by two reviewers. A meta-analysis was performed to examine the association between rs10937405, rs4488809 polymorphism at 3q28 and susceptibility to LC. Odds ratios (ORs) and 95% confidence intervals (95% CIs) were calculated. Heterogeneity and publication bias were also tested.

**Results:**

A total of 9 studies including 35,961 LC cases and 57,790 controls were involved in this meta-analysis. An overall random-effects per-allele OR of1.19 (95% CI: 1.14–1.25; P<10^−5^) and 1.19 (95% CI: 1.13–1.25; P<10^−5^) was found for the rs10937405 and rs4488809 polymorphism respectively. Similar results were also observed using dominant or recessive genetic model. After stratified by ethnicity, significant associations were found among East Asians (per-allele OR = 1.22, 95% CI: 1.17–1.27; P<10^−5^); whereas no significant associations were found among Caucasians for rs10937405. In the sub-group analysis by sample size, significantly increased risks were found for these polymorphisms in all genetic models. When analyzed according to histological type, the effects of rs10937405, and rs4488809 at 3q28 on the risk of lung cancer were significant mostly for lung adenocarcinoma.

**Conclusions:**

Our findings demonstrated that rs10937405-G allele and rs4488809-G allele might be risk-conferring factors for the development of lung cancer, especially for East Asian populations.

## Introduction

Lung cancer (LC) is the most common malignancy and the leading cause of cancer death worldwide, with an estimated 5-year survival rate of 15% [1]. The various histological forms of LC are typically divided into small cell lung cancer (SCLC) and non-small cell lung cancer (NSCLC) comprising adenocarcinoma and squamous tumors. Each of the LC types has different clinicopathological characteristics reflective of differences in carcinogenesis [2]. Despite much investigation, the causes are not yet fully understood. Epidemiological evidence suggests that exposure to tobacco-associated carcinogens is clearly implicated in its aetiology [3]. However, it has also been reported that only, 20% of smokers develop LC, suggesting that genetic variations and other environmental factors also play important roles in determining individual differences in LC susceptibility [4], [5].

Recently, spectacular advance was made in identifying susceptible genes involved in LC through genome-wide association strategy (GWAS). Genomic regions at chromosomes 15q25.1 (CHRNA5, CHRNA3 and CHRNA4) [6]–[8], 5p15.33 (TERT-CLPTM1L) [9], [10] and 6p21.33 (BAT3-MSH5) [9] have been identified to be associated with susceptibility to LC by GWAS in populations of European descent. However, studies have also shown the presence of genetic heterogeneity in LC susceptibility between populations of European descent and Asians [6]–[8]. A recent GWAS on lung adenocarcinoma risk in the Japanese and Korean populations identified a new locus, 3q28 (TP63) [11]. Subsequently, a significant but weaker association of 3q28 variations with lung adenocarcinoma risk was validated in Europeans [12]. To date, many case–control studies have been carried out to investigate the role of TP63 at 3q28 polymorphism in genetic susceptibility to LC among various populations. Genetic association studies can be problematic to reproduce due to insufficient power, multiple hypothesis testing, population stratification, source of controls, publication bias, and phenotypic heterogeneity. Therefore, we carried out a comprehensive meta-analysis on all eligible studies to estimate the overall LC risk of 3q28 polymorphism as well as to quantify the between-study heterogeneity and potential bias.

## Materials and Methods

### Literature Search Strategy

Genetic association studies published before the end of October 2013 on LC and polymorphisms within chromosome 3q28 were identified through a search of PubMed, Web of Science, EMBASE, and CNKI (Chinese National Knowledge Infrastructure) without language restriction. Search term combinations were keywords relating to the chromosome 3q28 (e.g., “3q28”, “rs10937405”, “rs4488809”, “tp63”) in combination with words related to LC (e.g., lung cancer’ or ‘lung carcinoma’ or ‘malignant lung neoplasm). In addition, studies were identified by a manual search of the reference lists of reviews and retrieved studies. All studies were carefully evaluated to identify duplicate data. Criteria used to determine duplicate data included study period, hospital, treatment information and any additional inclusion criteria.

### Eligible Studies and Data Extraction

To include relevant studies in this meta-analysis, the following criteria were used: (1) case-control or cohort studies, (2) assessing the association between 3q28 polymorphisms and LC risk, (3) identification of LC cases was confirmed histologically or pathologically, and (4) providing sufficient data to calculate the odds ratio (OR) with its 95 confidence interval (CI) and P-value. The major reasons for exclusion of studies were (1) overlapping data, (2) case-only studies, (3) family-based studies and review articles. If more than one article using the same data was published, only the study with the largest dataset was included into the meta-analysis.

Two investigators extracted information from all eligible publications independently according to the inclusion criteria listed above. For conflicting evaluation, an agreement was reached following discussion among all authors. For each study, the following characteristics were collected: first author’s name, year of publication, ethnicity, identification of cancer cases, histological type, age, sex, smoking status (never smokers or ever smokers), source of control groups (population-based controls and hospital-based controls), matching factors, Hardy-Weinberg equilibrium (HWE) status among controls, number of cases and controls, genotype frequency and genotyping methods. For studies including subjects of different ethnic groups, data were extracted separately and categorized as East Asians and Caucasians.

### Quality Assessment

For association studies with inconsistent results on the same polymorphisms, the methodological quality should be assessed by appropriate criteria to limit the risk of introducing bias into meta-analyses or systematic reviews. A procedure known as ‘extended-quality score’, has been developed to assess the quality of association studies. The procedure scores each paper categorizing it as having ‘high’, ‘median’ or ‘poor’ quality. Detailed procedure of the quality assessment was previously described [13].

### Statistical Methods

Deviation from HWE for controls was examined by χ^2^ tests. OR with 95% CIs was used to assess the strength of association between the 3q28 polymorphisms (rs10937405, and rs4488809) and LC risk. The per-allele OR of the risk allele was estimated. Then we estimated the risks of these polymorphisms on LC under dominant and recessive genetic models. Random-effects and fixed-effect summary measures were calculated as inverse-variance-weighted average of the log odds ratio. The results of random-effects summary were reported in the text because it takes into account the variation between studies. Heterogeneity across individual studies was calculated using the Cochran χ^2^ based Q test and I^2^ test followed by subsidiary analysis or by random-effects regression models with restricted maximum likelihood estimation [14], [15]. Generally, I^2^ values <25% correspond to no or little heterogeneity, values 25–50% correspond to moderate heterogeneity, and values >50% correspond to strong heterogeneity between studies. Sources of heterogeneity were investigated by stratified meta-analyses based on ethnicity, and sample size (No. cases ≥1000 or <1000). Ethnic group was defined as East Asians (i.e., Chinese, Japanese, and Korean), and Caucasians (i.e. people of European origin). In addition, ethnicity, sample size, age, sex and genotyping method was analyzed as covariates in meta-regression. The significance of the pooled OR was determined by Z test. Publication bias was assessed with the Egger test and Begg test [16], [17]. Sensitivity analysis was performed by removing each individual study in turn from the total and re-analyzing the remainder. The analysis was conducted using the Stata software version 10.0 (Stata Corporation, College Station, TX). All the P-values were for two-sided analysis and values of P<0.05 were considered statistically significant.

## Results

### Characteristics of Studies

Based on our search strategy, the primary screening produced 97 potentially relevant articles. 88 articles were excluded because they clearly did not meet the inclusion criteria or overlapping references. A total of 9 eligible studies [11], [12], [18]–[24] were included in the meta-analysis involving 35,961 LC cases and 57,790 controls. The literature selection process was showed in Figure S1. Among them, 23 data sets were identified for the rs10937405 polymorphism, including a total of 31,109 cases and 51,999 controls, and for the rs4488809 polymorphism 18 data sets were identified covering a total of 19,790 cases and 26,264 controls. The genotype distributions in the controls for all studies were consistent with HWE. Severn studies were given high quality, and two studies were given median quality. No ‘poor quality’ study was found. Characteristics of studies included in the current meta-analysis are presented in Table 1.

**Table 1:** Characteristics of the studies included in the meta-analysis.

Study	Year	Ethnicity	Cancer type	Control source	Match criteria	No. of cases/controls	Genotyping method	Quality
Miki [11]	2010	Japanese, Korean	Lung adenocarcinoma	Hospital	NA	2067/11030	SNP array, Invader assays	High
Wang [12]	2011	British	Lung cancer	Population	Sex and geographic region	3598/8166	SNP array	High
Hu [17]	2011	Chinese	Lung cancer	Hospital	Sex, sex and geographic region	8569/9416	SNP array, MassARRAY	High
Shiraishi [18]	2012	Japanese	Lung adenocarcinoma	Hospital	Geographic region	6009/12363	SNP array, Invader assays, TaqMan	High
Zhang [19]	2012	Chinese	Non-small cell lung cancer	Population	Age and sex	200/199	MassARRAY	Median
Lan [20]	2012	Chinese, Korean	Lung cancer	Hospital	Age and sex	4593/5450	SNP array, TaqMan	High
Hosgood [21]	2012	Chinese, Korean	Non-small cell lung cancer	Population	Geographic region	3422/3677	TaqMan	High
Timofeeva [22]	2012	European, American, Canadian	Lung cancer	Population,Hospital	Geographic region	7194/7179	SNP array	High
Hu [23]	2013	Chinese	Lung cancer	Population,Hospital	Geographic region	309/310	MassARRAY	Median

### Association of rs10937405 Polymorphism with Lung Cancer

**Figure 1:**
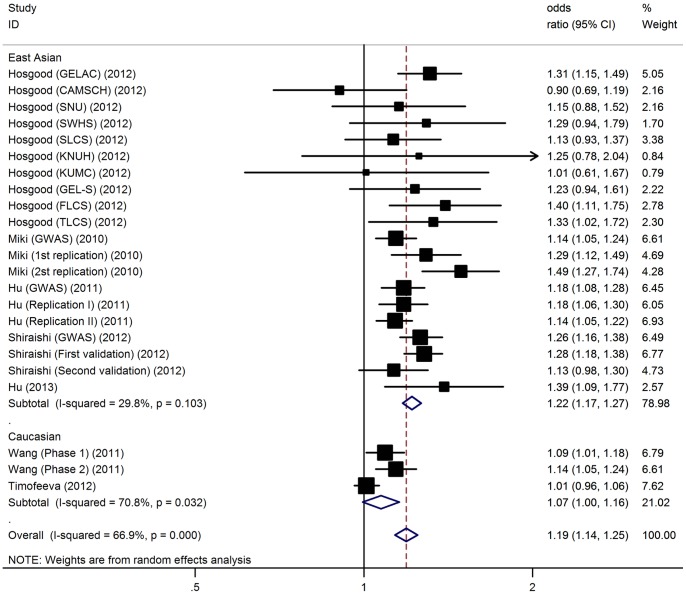
Forest plot for the meta-analysis of the association between rs10937405 polymorphism and lung risk.

Overall, there was evidence of an association between increased risk of LC and the rs10937405 polymorphism in different genetic models when all the eligible studies were pooled into the meta-analysis. Using random effect model, the summary per-allele OR of the rs10937405 G variant for LC was 1.19 [95% CI: 1.14–1.25, P(Z)<10^−5^, P(Q)<10^−5^; Figure 1], with corresponding results under dominant and recessive genetic models of 1.27 [95% CI: 1.15–1.40, P(Z)<10^−5^, P(Q)<10^−4^] and 1.25 [95% CI: 1.19–1.33, P(Z)<10^−5^, P(Q)<10^−4^], respectively. After adjusting for multiple testing using Bonferroni correction, all significant associations for rs10937405 under various genetic models remained. Significant heterogeneity was present among the 23 data sets (P<0.05). In meta-regression analysis, age (P = 0.26), sex (P = 0.11), sample size (P = 0.06), and genotyping method (P = 0.78), did not significantly explained such heterogeneity. By contrast, ethnicity (P = 0.001) was significantly correlated with the magnitude of the genetic effect.

When studies were stratified for ethnicity, significant risks were found among East Asians in all genetic model [G allele: OR = 1.22, 95% CI: 1.17–1.27; dominant model: OR = 1.26, 95% CI: 1.13–1.38; recessive model: OR = 1.28, 95% CI: 1.17–1.36]. However, no significant results were detected in the Caucasian populations [G allele: OR = 1.07, 95% CI: 1.00–1.16; dominant model: OR = 1.08, 95% CI: 0.99–1.21; recessive model: OR = 1.10, 95% CI: 1.00–1.21]. Subsidiary analyses of sample size yielded a per-allele OR for small studies of 1.27 [95% CI: 1.17–1.37, P(Z)<10^−5^] and for large studies of 1.16 [95% CI: 1.10–1.22, P(Z)<10^−5^; Table 2].

**Table 2:** Results of meta-analysis for rs10937405 polymorphism and lung cancer risk.

Overall andsubgroups analyses	No. of datasets	No. of case/control	G allele					Dominant model					Recessive model				
			OR (95% CI)	P(Z)	P(Q)^a^	I^2^ (%)	P(Q)^b^	OR (95% CI)	P(Z)	P(Q)^a^	I^2^ (%)	P(Q)^b^	OR (95% CI)	P(Z)	P(Q)^a^	I^2^ (%)	P(Q)^b^
Overall	23	31109/51999	1.19 (1.14–1.25)	<10^−5^	<10^−5^	75		1.27 (1.15–1.40)	<10^−5^	<10^−4^	52		1.25 (1.19–1.33)	<10^−5^	<10^−4^	57	
Ethnicity							<10^−4^					0.006					0.01
East Asian	30	20317/36654	1.22 (1.17–1.27)	<10^−5^	0.10	30		1.26 (1.13–1.38)	<10^−5^	0.08	20		1.28 (1.17–1.36)	<10^−5^	0.11	15	
Caucasian	3	10792/15345	1.07 (1.00–1.16)	0.07	0.03	71		1.08 (0.99–1.21)	0.08	0.01	49		1.10 (1.00–1.21)	0.05	0.09	9	
Sample size							0.003					0.005					0.001
<1000	12	3613/11974	1.27 (1.17–1.37)	<10^−5^	0.23	34		1.45 (1.15–1.83)	0.001	0.07	13		1.30 (1.19–1.42)	<10^−5^	0.66	0	
≥1000	11	27496/40025	1.16 (1.10–1.22)	<10^−5^	<10^−4^	48		1.20 (1.12–1.29)	<10^−5^	0.62	0		1.22 (1.15–1.29)	<10^−5^	0.10	11	

a Cochran’s chi-square Q statistic test used to assess the heterogeneity in subgroups.

b Cochran’s chi-square Q statistic test used to assess the heterogeneity between subgroups.

In subgroup analyses by histological types of LC, we found that the rs10937405 polymorphism was significantly associated with lung adenocarcinoma [G allele: OR = 1.26, 95% CI: 1.21–1.30, P(Z)<10^−5^] and squamous cell carcinoma [G allele: OR = 1.14, 95% CI: 1.06–1.22, P(Z)<10^−4^], while no significant associations were detected for small cell carcinoma (Table 3). We investigated the relationship between the polymorphism and known environmental tobacco exposures. The effect of environmental tobacco smoke was similar for never smokers with per-allele OR of 1.27 (95% CI: 1.21–1.33), compared to ever smokers (OR = 1.26, 95% CI: 1.18–1.34). Furthermore, we stratified the included studies by sex. Similarly, statistically significant results were also observed in both male and female (Table 3).

**Table 3:** Per-allele OR and 95% CI association between 3q28 common variations and lung cancer risk by histologic type, smoking status, and sex.

Variants	Risk allele	Subgroup	No. data sets	No. of case/control	Risk allele				
					OR (95% CI)	P(Z)	P(Q)^a^	I^2^ (%)	P(Q)^b^
rs10937405	G	Adenocarcinoma	18	11503/35238	1.26 (1.21–1.30)	<10^−5^	0.68	0	<10^−5^
		Squamous cell carcinoma	3	1442/11845	1.14 (1.06–1.22)	<10^−4^	0.60	0	
		Small cell carcinoma	2	1505/8166	1.03 (0.94–1.13)	0.54	0.54	0	
		Never smoker	13	6784/13125	1.27 (1.21–1.33)	<10^−5^	0.50	0	0.81
		Ever smoker	3	3305/12572	1.26 (1.18–1.34)	<10^−5^	0.60	0	
		Male	3	3244/14390	1.27 (1.21–1.33)	<10^−5^	0.45	0	0.80
		Female	13	6856/12479	1.26 (1.18–1.34)	<10^−5^	0.91	0	
rs4488809	G	Adenocarcinoma	14	9621/20504	1.19 (1.10–1.29)	<10^−5^	<10^−5^	72	0.005
		Squamous cell carcinoma	3	3381/13292	1.08 (0.87–1.34)	0.46	0.01	35	
		Small cell carcinoma	1	782/9416	1.05 (0.94–1.16)	0.36	NA	NA	
		Never smoker	12	11543/14710	1.15 (1.05–1.27)	0.003	<10^−5^	69	0.89
		Ever smoker	2	5041/3833	1.21 (1.14–1.29)	<10^−5^	0.99	0	
		Male	1	5864/6214	1.25 (1.18–1.32)	<10^−5^	NA	NA	0.19
		Female	12	10720/12329	1.15 (1.05–1.27)	0.003	<10^−5^	82	

a Cochran’s chi-square Q statistic test used to assess the heterogeneity in subgroups.

b Cochran’s chi-square Q statistic test used to assess the heterogeneity between subgroups.

### Association of rs4488809 Polymorphism with Lung Cancer

**Figure 2:**
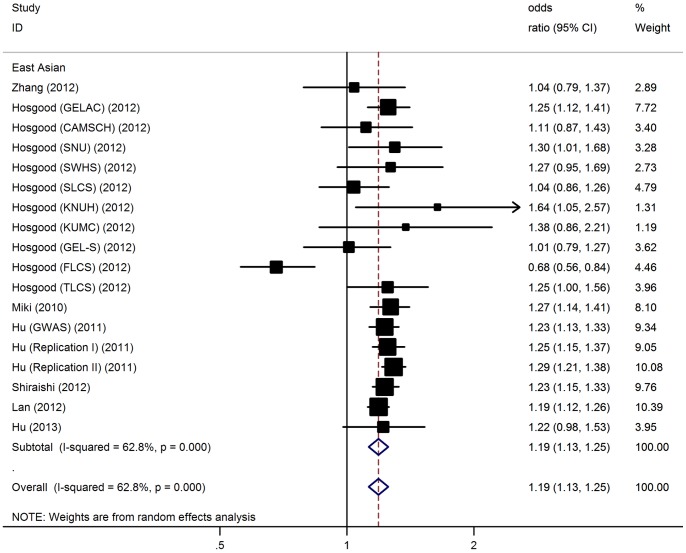
Forest plot for the meta-analysis of the association between rs4488809 polymorphism and lung risk.

For LC risk and the rs4488809 polymorphism, our meta-analysis gave an overall OR of 1.19 (95% CI: 1.13–1.25, P<10^−5^; Figure 2) with statistically significant between-study heterogeneity (P<10^−5^). Significantly increased LC risks were also found using dominant [OR = 1.24, 95% CI: 1.11–1.38, P(Z)<10^−5^; P(Q)<10^−4^] and recessive model [OR = 1.25, 95% CI: 1.14–1.37, P(Z)<10^−5^; P(Q) = 0.01]. Since all included studies concerning rs4488809 polymorphism were conducted in East Asian populations, we then conducted subgroup analysis by sample size. In the stratified analysis by sample size, significant risks were found among larger studies in all genetic models (G allele: OR = 1.24, 95% CI: 1.20–1.28, P<10^−5^; dominant model: OR = 1.39, 95% CI: 1.31–1.48, P<10^−5^; recessive model: OR = 1.30, 95% CI: 1.22–1.37, P<10^−5^) without significant between-study heterogeneity (P>0.05 for all genetic models). Similar significant associations were also observed among small studies using dominant and recessive genetic models (Table 4). After adjusting for multiple testing using Bonferroni correction, all significant associations for rs4488809 remained.

**Table 4:** Results of meta-analysis for rs4488809 polymorphism and lung cancer risk.

Overall and subgroupsanalyses	No. of data sets	No. of case/control	G allele					Dominant model					Recessive model				
			OR (95% CI)	P(Z)	P(Q)^a^	I^2^ (%)	P(Q)^b^	OR (95% CI)	P(Z)	P(Q)^a^	I^2^ (%)	P(Q)^b^	OR (95% CI)	P(Z)	P(Q)^a^	I^2^ (%)	P(Q)^b^
Overall	18	19790/26264	1.19 (1.13–1.25)	<10^−5^	<10^−5^	63		1.24 (1.11–1.38)	<10^−5^	<10^−4^	58		1.25 (1.14–1.37)	<10^−5^	0.01	70	
Sample size							0.003					0.14					0.22
<1000	11	2741/3042	1.12 (0.97–1.28)	0.11	0.001	56		3.28 (2.82–3.82)	<10^−5^	0.05	28		2.67 (2.20–3.25)	<10^−5^	0.02	68	
≥1000	7	17049/23222	1.24 (1.20–1.28)	<10^−5^	0.73	0		1.39 (1.31–1.48)	<10^−5^	0.38	14		1.30 (1.22–1.37)	<10^−5^	0.81	0	

a Cochran’s chi-square Q statistic test used to assess the heterogeneity in subgroups.

b Cochran’s chi-square Q statistic test used to assess the heterogeneity between subgroups.

In subgroup analyses by histological types of LC, we found that the rs4488809 polymorphism was significantly associated with lung adenocarcinoma [G allele: OR = 1.19, 95% CI: 1.10–1.29, P(Z)<10^−5^] However, no significant associations were detected for squamous cell carcinoma or small cell carcinoma (Table 3). Associations of the polymorphism with LC risk were observed both in ever smokers and never smokers and both male and female (Table 3).

In meta-regression, sample size (P = 0.02) explained a large part of the heterogeneity, whereas sex distribution (P = 0.29), mean age (P = 0.14), and genotyping method (P = 0.31) explained little heterogeneity.

### Sensitivity Analyses and Publication Bias

A single study involved in the meta-analysis was deleted each time to reflect the influence of the individual data-set to the pooled ORs, and the corresponding pooled ORs were not qualitatively altered (Figure S2 and S3). The shape of the funnel plots was symmetrical for these polymorphisms (Figure S4 and S5). The statistical results still did not show small study effects in these studies rs4488809 (Egger test, P = 0.30) and rs10937405 (Egger test, P = 0.06).

## Discussion

GWAS have led to the identification of multiple new genetic variants associated with lung cancer risk. Most of these lung cancer GWAS and replication studies have been conducted in European populations [6]–[10] and to a lesser extent in East Asians [11], [18], [19]. However, there are significant differences in allele frequencies and the prevalence of lung cancer among different populations. It is, therefore, important to quantitatively assess the effects of the GWAS-identified markers in different ethnic populations and explore potential heterogeneity of published data. Meta-analysis is a means of increasing the effective sample size under investigation through the pooling of data from individual association studies, thus enhancing the statistical power of the analysis for the estimation of genetic effects [25]. This is the first comprehensive meta-analysis, which comprise a total of 35,961 cases and 57,790 controls from 9 case–control studies, examining the association of two commonly studied polymorphisms of 3q28 (rs10937405, and rs4488809) with lung cancer risk. Our results demonstrated that rs10937405-G allele, and rs4488809-G allele is a risk factor for developing lung cancer.

In the stratified analysis by ethnicity, significant associations were found in East Asians for rs10937405 polymorphism in all genetic models; while no associations were found in Caucasians, suggesting a possible role of ethnic differences in genetic backgrounds and the environment they lived in [26]. In fact, the distribution of the risk G allele varies extensively between different races, with a prevalence of ∼70% among East Asians [19]–[22], and ∼55% among Caucasians [12], [23]. Thus, failing to identify any significant association in Caucasian populations could be due to substantially lower statistical power caused by the relatively lower prevalence of G allele of rs10937405. Therefore, additional studies are warranted to further validate ethnic difference in the effect of this polymorphism on lung cancer risk. Furthermore, study design or small sample size or some environmental factors may affect the results. It is possible that variation at this locus has modest effects on lung cancer, but environmental factors may predominate in the progress of lung cancer, and mask the effects of this variation. Specific environmental factors like lifestyle and smoking that have been already well studied in recent decades [1]. In addition, different populations usually have different linkage disequilibrium patterns. A polymorphism may be in close linkage with another nearby causal variant in one ethnic population but not in another. These polymorphisms may be in close linkage with different nearby causal variants in different populations. As for rs4488809, only studies conducted among East Asians were available currently, further studies including a wider spectrum of subjects to investigate the role of this variant in different ethnic populations will be needed to confirm our results.

When analyzed according to histological type, the effects of rs10937405, and rs4488809 at 3q28 on the risk of lung cancer were significant mostly for adenocarcinoma. However, adenocarcinoma accounts for about 50% of all histological types of lung cancer [11]. The reason for the observed tumour-specific difference in the risk conferred by these polymorphisms is unknown. However, different carcinogenic processes may be involved in the genesis of various tumour types because of the presence of functionally different 3q28 polymorphisms. Hence, future studies should use homogeneous cancer patients. When stratified by smoking behavior and sex, these SNPs tended to have similar OR for females and males, never smokers and ever smokers.

While it will be challenging to identify the precise mechanism by which 3q28 variation affects lung cancer development, accumulation of DNA damage and lack of response to genotoxic stress is recognized to contribute to lung carcinogenesis. TP63 (also known as p63) is a member of the tumor suppressor P53 gene family, which is pivotal to cellular differentiation and responsiveness to cellular stress [27]. Exposure of cells to DNA damage leads to induction of TP63 and both isoforms have the ability to transactivate TP53 target genes, hence impacting on cellular responsiveness to DNA damage [28], [29]. TP63 is expressed mainly in two isoforms, the TA and N-terminal-truncated (ΔN) forms, and its biological role appears to be more complex than that of a classic tumor suppressor [30]. TAp63 isoforms appears to be highly transcriptionally potent and is essential for maintaining adult epidermal stem cells [31], cell proliferation, differentiation and survival upon NF-κB activation [32]. TP63 overexpressed and amplified might increase invasiveness and aggressiveness of cells in normal or hyperplastic epithelial cells [33], [34]. Accumulation of DNA damage and lack of response to genotoxic stress contribute to an earlier step in carcinogenesis. Because possible candidate SNPs are located in intron 1 of TP63, which encodes TAp63 isoforms, these results suggest that one or more of these SNPs may have a functional role in the regulation of TP63 gene expression.

The strengths of this study include the very large sample size, no deviation from HWE, and the high quality of the qualified studies. However, our current study should be interpreted with several technical limitations in mind. Firstly, the vast majority of white subjects in the study are of East Asian descent, and statistical power for analyses in other ethnicities is limited. Because the sample size was relatively smaller for Caucasian studies, the main conclusions from this manuscript are based on analyses among white East Asian populations. Future studies including larger numbers of Caucasian or African are necessary to clarify the consistency of findings across ethnic groups. Secondly, our results were based on unadjusted estimates, while a more precise analysis should be conducted if individual data were available, which would allow for the adjustment by other covariates including age, family history, environmental factors and lifestyle. Thirdly, the subgroup meta-analyses considering associations between the two polymorphisms and smoking behavior, sex, as well as between various histologic type of lung cancer were performed on the basis of a fraction of all the possible data to be pooled, so selection bias may have occurred and our results may be overinflated. Nevertheless, the total number of subjects included in this part of the analysis comprises the largest sample size so far.

Despite these limitations, our meta-analysis showed that rs10937405, and rs4488809 polymorphism at 3q28 might be risk-conferring factor for the development of lung cancer, especially for East Asian populations. Larger studies of different ethnic populations, especially with detailed individual information, are needed to confirm our findings. Since it is unlikely that genetic variation alone will explain the lung cancer burden, further research is needed to identify environmental exposures and gene–environment interactions in these populations. Further, the functional genetic variants and mechanisms underpinning this association will require additional studies.

## Supporting Information

Figure S1 **Study selection process.**
(TIF)

Figure S2 **Result of sensitivity analyses for 3q28-rs10937405 polymorphism and LC risk.**
(TIF)

Figure S3 **Result of sensitivity analyses for 3q28** ***-*** **rs4488809 polymorphism and LC risk.**
(TIF)

Figure S4 **Begg’s funnel plot of 3q28-rs10937405 polymorphism and lung cancer risk.**
(TIF)

Figure S5 **Begg’s funnel plot of 3q28** ***-*** **rs4488809 polymorphism and lung cancer.**
(TIF)

Checklist S1 (DOC)
