# Supplementary figures and images for: Quantitative Assessment of the Influence of TP63 Gene Polymorphisms and Lung Cancer Risk: Evidence Based on 93,751 Subjects
Source: PLoS One. 2014 Jan 23;9(1):e87004. doi: 10.1371/journal.pone.0087004 (PMC3900682; doi:10.1371/journal.pone.0087004)

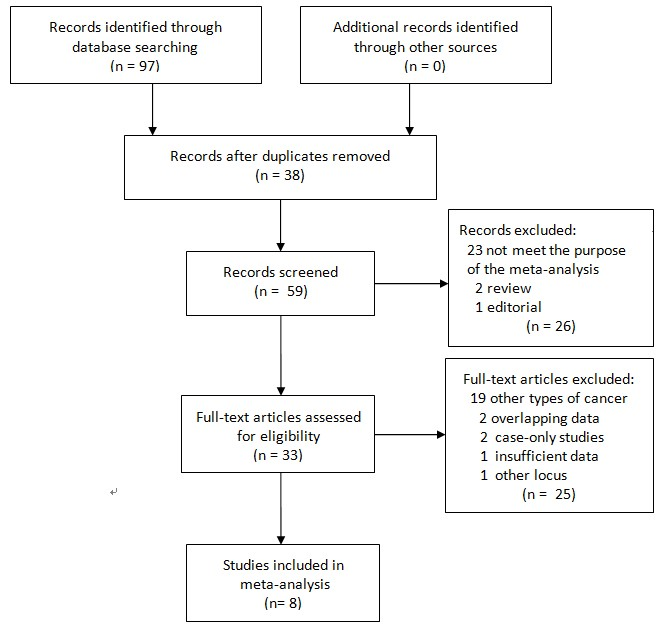

Supplement: Figure S1 — Study selection process. (TIF) [file pone.0087004.s001.tif]

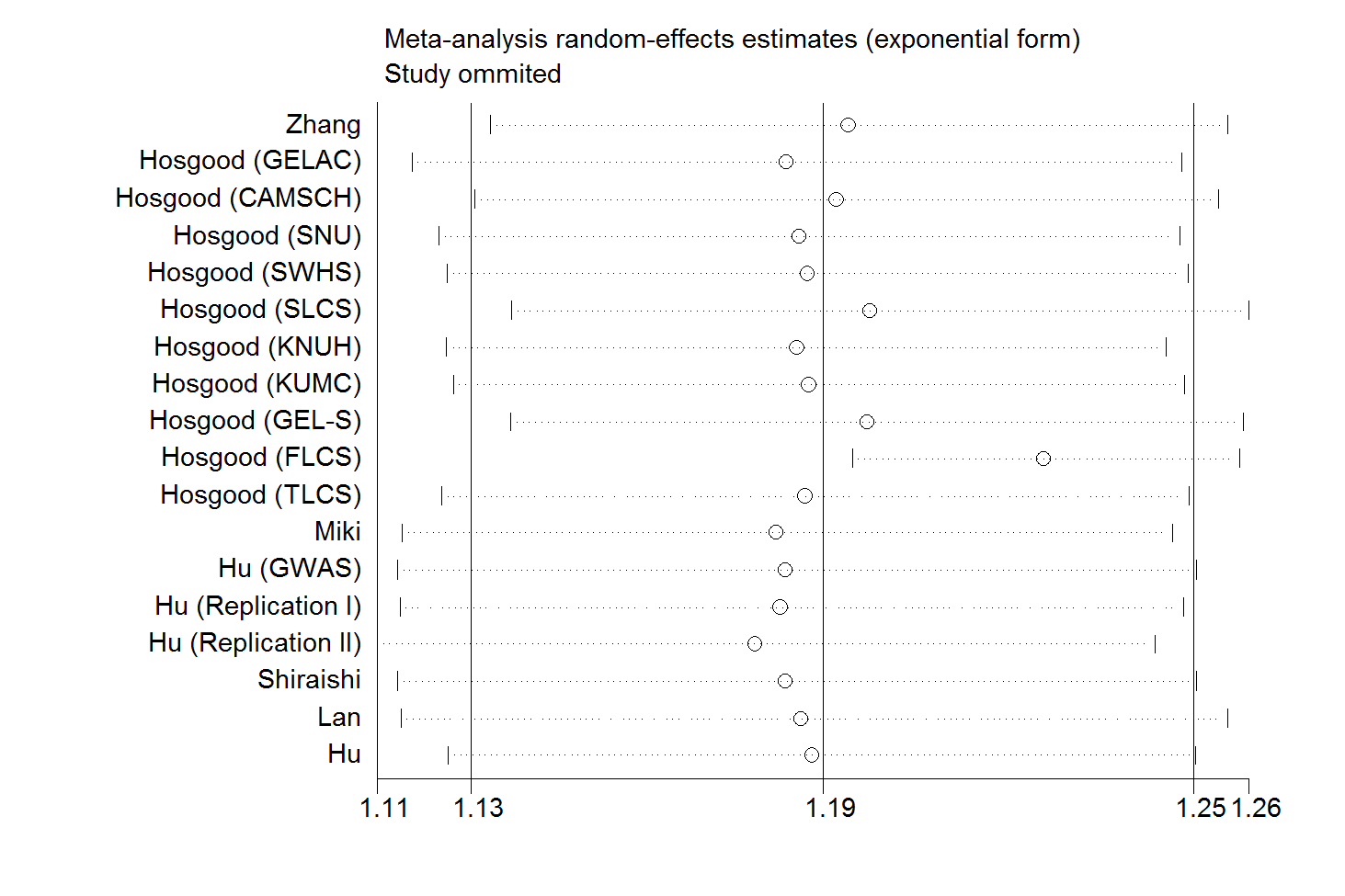

Supplement: Figure S2 — Result of sensitivity analyses for 3q28-rs10937405 polymorphism and LC risk. (TIF) [file pone.0087004.s002.tif]

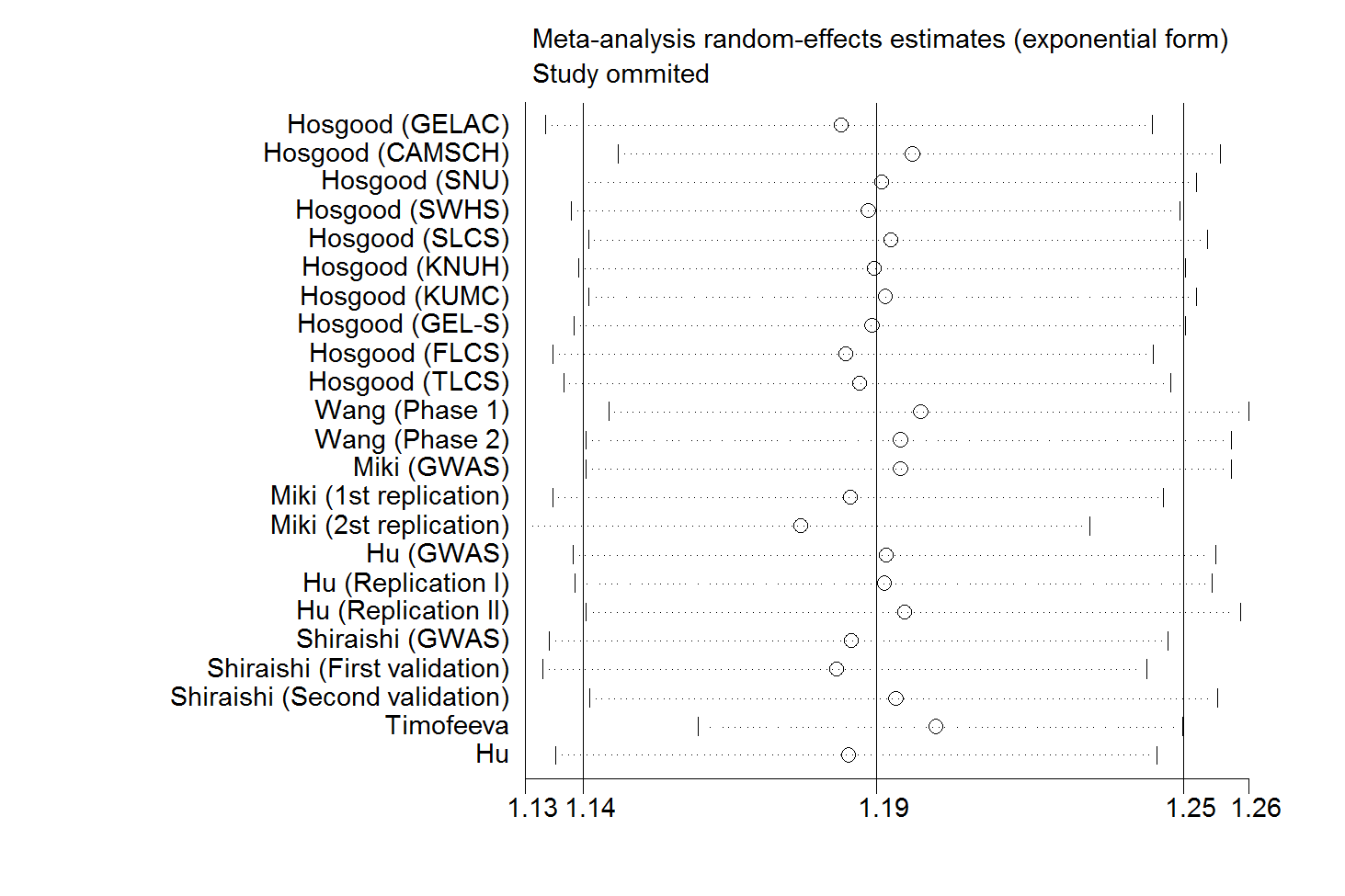

Supplement: Figure S3 — Result of sensitivity analyses for 3q28 - rs4488809 polymorphism and LC risk. (TIF) [file pone.0087004.s003.tif]

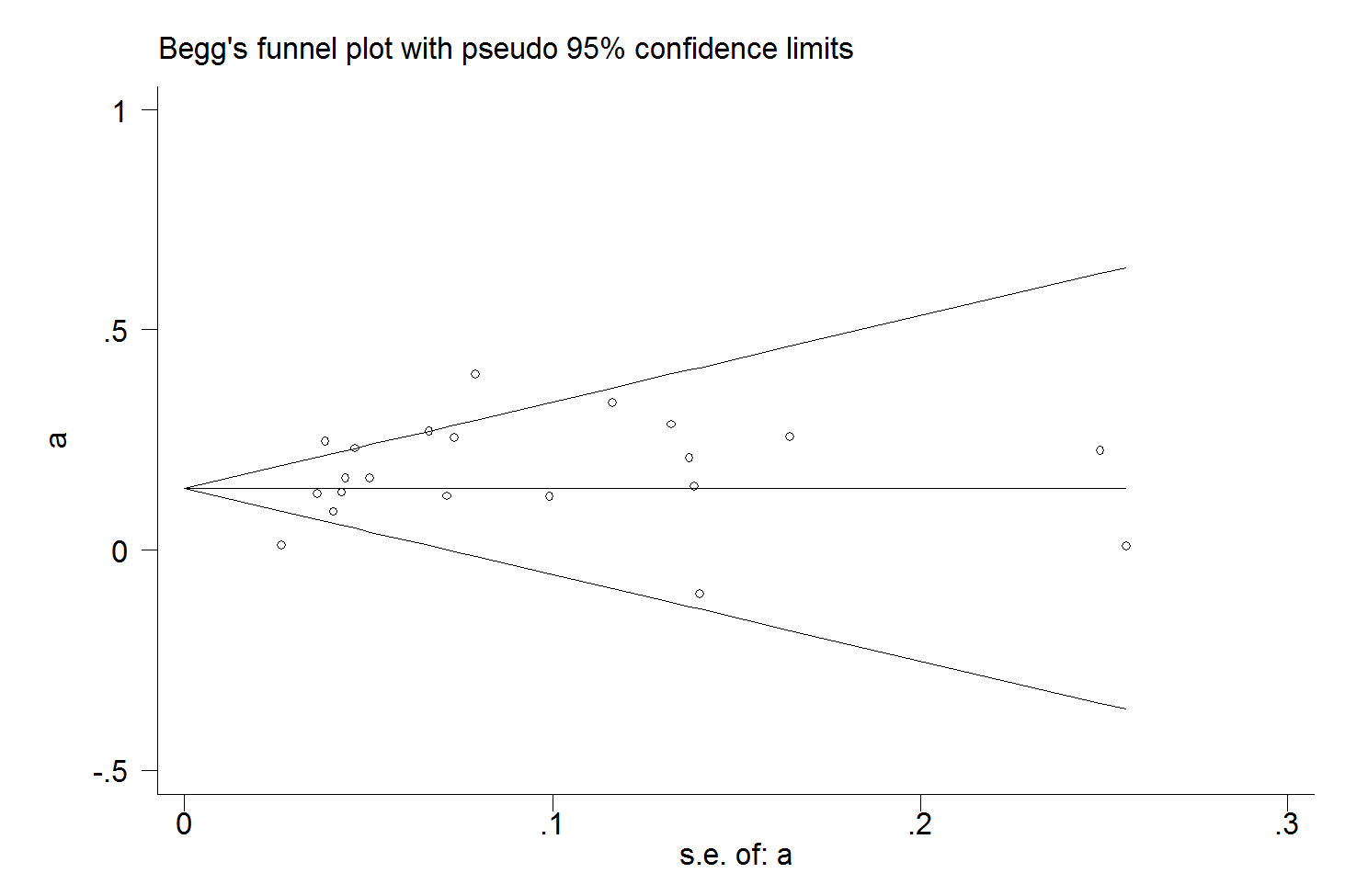

Supplement: Figure S4 — Begg’s funnel plot of 3q28-rs10937405 polymorphism and lung cancer risk. (TIF) [file pone.0087004.s004.tif]

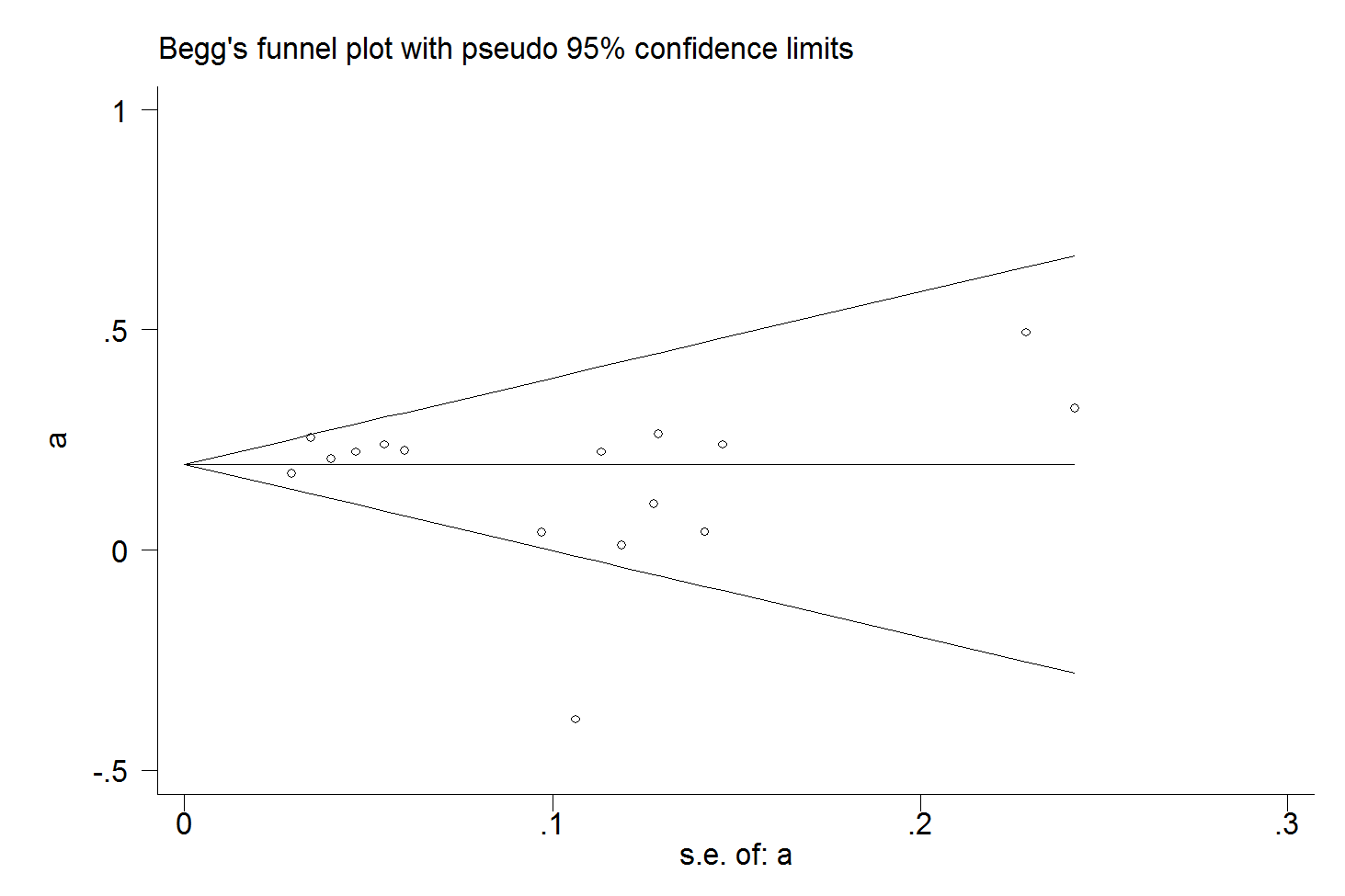

Supplement: Figure S5 — Begg’s funnel plot of 3q28 - rs4488809 polymorphism and lung cancer. (TIF) [file pone.0087004.s005.tif]
